# Care for the wounded at Vukovar Medical Center: new task for two general practitioners

**DOI:** 10.3325/cmj.2011.52.95

**Published:** 2011-02

**Authors:** Ivanka Mihajlović, Blanka Vagenhofer

**Affiliations:** Medical Center, Vukovar, Croatia

## Ivanka Mihajlović

I was born in Vukovar in 1960. I graduated from the Zagreb University School of Medicine and did my internship in Vukovar. Before the war, I worked as a general practitioner in an outpatient clinic for five years. In mid-August 1991, I was transferred to the Hospital Emergency Unit. During the last two months, I took care of wound dressing and timely medication of the wounded.

## Blanka Vagenhofer

I was born in Vukovar in 1960. I graduated from the Zagreb University School of Medicine, Osijek Branch, and did my internship in the Vukovar Medical Center. Before the war, I worked in the pediatric office in Borovo Naselje. Around mid-August, frequent alarms and attacks made it impossible for us to normally proceed with our work, so care for sick children was organized in the hospital. There, I continued to work at the Intensive care Unit. It was my first contact with this field of medicine. At the same time I had to learn and daily cope with practical problems. Several anesthesiologists had left the hospital and those who had arrived to replace them were continuously working in operating theaters. A colleague of mine, in her 2nd year of residence, and I were in charge of intensive care patients. Four to five nurses worked on our shift.

## The beginning of war

The war began gradually, from June of 1991. The everyday rhythm would temporarily be disturbed by machine-gun fire and occasional explosions. Dr Vesna Bosanac, a pediatrician, was appointed the head of the Hospital. She organized dislocated medical units for patient care. Operating theaters were organized in the cellar of the Eltz's castle, which was destroyed before long. A field hospital was established in the shelter in Bogdanovci, and the ward for the lightly wounded in the *Borovokomerc* shelter. Dr Bosanac also had the hospital anti-atomic bomb (AA) shelter adapted for patient admission. The hemodialysis patients had previously been transferred to Rovinj in Istria, because the way to Osijek was cut off in May 1991.

At the beginning of September, the aggression on Vukovar intensified. We could not leave the hospital anymore. Duty hours were organized so that 24-hour work was followed by 24-hour rest. General practitioners worked during the day when necessary, rested during the night, slept in various rooms in the old building's first floor. The plaster room and the x-ray room were adapted in two operating theaters. Accessory operating theater was left in the same location, while two rooms were adapted for admission of surgical patients. Halls of the Internal Medicine (the 2nd floor) and Surgery (the 1st floor) Wards and the underpass connecting the old and the new buildings, became large patient rooms. Medicines, water from small cisterns and food for patients were dispensed at the entrance into the AA shelter. In one of the rooms (with three iron bunk beds each), infants in incubators, newborns (16 babies were born), pregnant women and healthy children of the personnel, were accommodated together. The Intensive Care unit was also organized in the AA shelter.

## Attacks on the hospital – sequence of events

The old and the new wings with inpatient wards, as well as all other hospital premises, were directly shelled from multiple rocket launchers, mortars, and tanks. At first, certain parts of the hospital were intentionally targeted to be destroyed. The consequences of grenade explosions were also visible in the hospital yard, despite the fact that a large white sheet with a red cross in the middle of it was stretched over the AA shelter after the Red Cross signs on the hospital roof had been destroyed. The holes in the walls and broken windows were stuffed with bed-sides, cardboard, and plastic sheets.

In the beginning, the wounded were stationed in the first-floor corridors and moved down to the underpass during alarms. Soon, they had to stay down all the time. The first floor was only used when the basement and underpass were overcrowded. At the beginning of October, 80 wounded were examined in a single day. The hall of the admission room was flooded with blood – it was swept away by a broom. On that occasion, 40 wounded were placed at the first floor. As grenades then hit just that part of the hospital, they were quickly removed to the lower floor. They lay in twos in beds, on the floor, under the beds... Practically, the beds were hardly accessible. The first-floor walls swayed during attacks and threatened to crush down.

We were expecting the first convoy for evacuation. It did not come. When the second convoy arrived, the wounded were transferred in the rain to the trucks covered with tarpaulin, with polystyrene foam on the floor. For as long as it was possible, lightly wounded were transferred to the ward in the *Borovokomerc* shelter, but this connection was soon cut off. All the wounded had to stay in the hospital. Toward the end of October, the hospital was hit by 250-kg bombs. The building quivered, the walls swayed as on the waves, the door-posts and window sashes were thrown out. In the ground-floor, glass from the compartment walls showered upon the patient beds in the hall. Four sacks of glass were collected there. One bomb fell along the outer part of the building, setting two cars on fire (an ambulance and a private car). Through broken windows of the Surgical and Internal Medicine Wards, black smoke poured out. The patients in the ground-floor drew their infusion catheters out, cried for help, jumped, and those who could not walk crept out of their beds. Another bomb broke through the loft, the ceiling of the Surgery and Internal Medicine Wards, and the ground-floor to fall unexploded on the bed, between the legs of a patient (P.V.). The patient, who had upper arm injury jumped from his bed. Extremely astonished, a nurse asked where that oxygen bomb came from, right onto the bed (she did not even realize at first what it actually was!). In the Intensive Care Unit in the AA-shelter, a “strong air wave” blew the hair in our faces. Later we saw the sky through a hole in the ground-floor ceiling.

Over the last 15 days, the Hospital was daily exposed to fierce several-hour, ceaseless attacks. In the AA-shelter, the patient beds rocked, the boxes we sat upon swayed. During an attack, pieces of concrete fell down in front of the shelter entrance and one lightly injured a patient. It was the only injury inflicted upon the patients during all those attacks on the hospital.

Two days before the fall of Vukovar, the premises of the Police Station and the Court, which partially protected our building, were set on fire and demolished. The hospital was now a plainly exposed target for the Yugoslav Federal Army (YFA) artillery from across the river Danube. We expected them to set the hospital on fire. The wounded were not brought in anymore. Later we learned they had all been referred to the Borovo Naselje shelter, because all the ways to the hospital were cut off. Conditions in that shelter, however, were inadequate for proper care of the severely wounded.

## The end

When Vukovar fell, YFA “evacuated” 300 wounded along with some thousand civilians, and transferred them to Borovo Selo, a village occupied by the Serbian paramilitary forces. The wounded were not escorted by Red Cross members. We know nothing about their fate. Only one nurse has managed somehow to reach Zagreb.

At that time, before the fall of Vukovar, Dr Bosanac contacted major Mrkšić, commander of the Vukovar YFA Garrison, asking him to protect the wounded and the hospital staff from the possible insults by the Serbian paramilitary. She came back and told us about a possible evacuation on Sunday, at 2 am On 20 November 1991, the YFA officers were the first to walk through the hospital door accompanied by the former hospital porter, a Serb, now in military uniform. The Hospital was surrounded by YFA soldiers who forbade anybody to get out. They demanded all the rooms to be searched through and all arms to be given up. They took complete medical records on all the patients, wounded and dead. Nine YFA soldiers treated at the Hospital were immediately taken away. Soldiers walked around with Scorpion guns lifted high in their hands. They did not contact us or made any comments. They were quite correct. We do not know how they behaved toward the wounded, because we were not allowed to stay with our patients. The day passed quietly.

The next evening, the cast-makers M.M. and Z.V., who had taken photographs of the hospital, were taken away, and so was the court inspector. They were told to give all the photo material up.

The following morning, Dr Lj. Š., anesthesiologist, asked us all to gather in the cast-room at 7:30. We, the doctors, and the nurses were separated from other hospital staff. YFA officer Veselin Šljivančanin gave a political speech, explaining that they had come to liberate us. He acquitted Dr Bosanac of her duty as the head and appointed a new director. During that time, a part of the wounded were taken out through the emergency exit. We were not allowed to check who was left and who was transferred to the YFA barracks.

We had four options: to go to Šid or Novi Sad in Serbia or to Zagreb via Đakovo or to stay in Vukovar. Five physicians stayed in Vukovar. We did not see Drs V. Bosanac and our surgeon J. Njavro anymore. We brought only the most necessary personal articles along and left the shelter with about 180 wounded. We were crowded in buses and army transporters, and escorted by the Red Cross and European Community Monitoring Mission members; we saw them within the hospital premises for the first time. At that moment, we did not know that they had been forbidden by YFA to enter the hospital the day before.

Patient lists with diagnoses were prepared the day before. Seven doctors and three nurses were cut off in Borovo. Dr Bosanac told us she would wait for their evacuation since she could not leave them there and just go. We did not see her anymore.

We left the ruins of Vukovar. Three vehicles of our convoy were lost on the way, their YFA drivers “got lost.” Later on, they re-joined us. In Bijeljina, in Bosnia, after we had passed Sremska Mitrovica, a group of some 200 people, civilians and Serbian paramilitaries (the so-called Arkan's Chetniks) waited for us and halted the convoy. The army transporters and buses stopped, we had to go out, where we were insulted, attacked, threatened, in front of the YFA and local police who just stood aside and watched. A Serbian paramilitary soldier entered the bus and yelled at an old man that he was Arkan's Chetnik, another one recognized one of the wounded and threatened he could kill him on the spot. These were incidents on our way back. As advised by the EC officials, we returned to the buses. Our ambulances and buses were stoned. We headed for Đakovo and then for Zagreb.

## Working conditions in hospitals

### Electrical current, water, heating

We worked in the Medical Center without electricity, water, and heating. Electric generators were demolished and only one was left. In the evening, candles and oil-lamps were used. On several occasions, surgical procedures were performed under the light of wax-candles.

Water was brought in tank trucks from Borovo. They were demolished soon and three firemen were killed. The wash-room was hit and ceased functioning a week before the end. Hospital washing was rinsed in cold water and dried in the corridors. During the last three weeks, water was supplied from the nearby wells. It had to be hyperchlorinated before use. One liter of water was provided per person daily or for per os tablet administration for 20 patients. Water from the heating system was also let out and rain water collected. Water distillation was carried out in the house of a civilian who had a brandy-distillation device. After two days, the YFA learned about it and destroyed the house 4 days before the fall of Vukovar. On the last day, we washed our faces in grayish-brown water.

The engine-room worked for 2-3 days. Then it was hit by a grenade, killing the man working there (R.I.). The sterilization room, located at the ground-floor, was shelled on several occasions. The remaining sterilizers were moved to another, smaller room, where they were hit again. Only one dry sterilizer was left. Surgical sets were washed in coldwater.

### Kitchen and catering

The kitchen, located outside the old hospital building, was hit at the very beginning. Then it was moved to the Ophthalmology Outpatient Office at the ground-floor, where two wood-stoves were put into function. It was systematically shelled at lunch time and around 5 pm, so that the cooks frequently had to leave the room. Three meals were prepared daily for all the people in the hospital: tea and biscuits for breakfast, and warm meal for lunch and dinner, almost always meat with rice, potatoes, or paste. Bread was regularly brought from the Borovo bakery, which also was frequently shelled. While repairing the bakery roof perforated by a grenade, V.V., a civilian, lost his hand.

During the last two weeks, ie, since the communication with Borovo had been cut off, the civilians made bread and small cakes without yeast at their homes. The patients were given a slice and we a half a slice each.

### Intensive Care Unit

Intensive Care Unit was located in one of the AA shelter attack-proof rooms. The room had two doors; the children played there, support personnel involved in dispensing food and water frequently went by, just like those needing medicines from the pharmacy. It was all but quiet, protected from grenades, and construction collapse. During the night, it was stuffy as there was no ventilation.

The initial amounts of infusion solution and drugs, along with appropriate laboratory diagnosis, allowed adequate examinations and proper follow-up of the patients. With time, the number of severely wounded requiring intensive care increased. The patients were frequently kept at the Intensive Care Unit of 36 m^2^ for only a short time, just to stabilize their blood pressure and pulse. The turnover of patients there was high, 2-3 times during a single day. Occasionally, a decision on moving from the Intensive Care Unit had to be made instantaneously. Selection of the patients was practically impossible, so they were moved to other areas, but accompanied with intensive care lists. The four places intended for such a purpose accommodated 6, and toward the end 8-9 patients.

Surgical procedures had to be delayed by 5-6, occasionally 24 hours. Oxygen and oxydul were used for anesthesia. Extremity amputations were performed under spinal anesthesia. It was impossible to operate on all patients under general anesthesia. We had enough anesthetics for iv. administration. There was only one respirator, it was always in operation! During that time, ambu-masks were used for manually assisted pre- and postoperative ventilation of other patients.

## Diagnosis and treatment

In the beginning, each wounded person was completely diagnostically examined on admission. Later, the laboratory was demolished and the x-ray rooms destroyed. The voltage was too weak and x-ray machines were only used for the most severely wounded.

Over the last two months, only erythrocytes and hematocrit could be determined. Blood glucose was determined with diagnostic test strips. Only one cell-counter was left. Sufficient amounts of donated medical materials were available at hospital’s pharmacy. Solutions for liquid, electrolyte and nutrient substitution were supplied in sufficient amounts, and so were also catheters, needles, and other consumables.

Initially, the treatment was performed according to peace-time protocols, then according to the Medical Corps doctrine. Later on, when the blockade began, the supplies started to run out. Drugs and medical material from the three town pharmacies (Mitnica, Borovo, and the center) were transferred to the hospital’s pharmacy. Toward the end, antibiotics for parenteral administration ran out, we had only those for per os use. Freezers did not work due to inadequate voltage, so plasma and blood derivates could not be stored. Blood was obtained from donors – civilians and hospital personnel. We also went short of plastic bags for blood storage.

## The wounded

All the wounded were equally treated, regardless of their religion or nationality. Civilians comprised two thirds of them. Children were also wounded, the youngest patient aged 6.5 months – she was injured by a grenade. She survived and was evacuated with us. We also treated nine YFA soldiers. Three of them were placed close to Dr Bosanac's office, with a special guard. During one of the attacks, the watchmen rushed into the room together with the wounded and took those YFA members out. Then, the outer wall of the room fell down when hit by a grenade. These soldiers were even much better treated than the Croatian guardsmen or civilians: they were given more food and cigarettes.

One YFA soldier was all alone, without his parents and brother, who had been killed in a traffic accident, and his grandmother had died during the war. He died from gaseous gangrene, after high above-knee amputation. He did not even wake up from anesthesia. There were several wounded who remained paraplegic. We believed the condition was transitory, consequential to the posttraumatic edema. There was no overt spine injury. Unfortunately, neither there were any signs of reinnervation.

Two YFA soldiers died at the Intensive Care Unit; one from a severe head injury. He was admitted with a pulse of 40 bpm, unconscious, with hopeless prognosis. We kept him on the only respirator we had for 7 days. The other, mentioned above, died from gaseous gangrene. Before that, he was given six bottles of blood, although the others always received one bottle each, regardless of indications. Gaseous gangrene caused three deaths, and six patients had to undergo amputation; one of them was an Orthodox priest.

The injuries were quite bizarre and their complications unpredictable. Thus, we recorded a bulbus protrusion after a retrobulbar passage of a piece of shrapnel, or a part of the face or body blown away, with the rest completely intact. On surgery, a small entrance wound would reveal a large wound canal. We encountered mostly explosive wounds, gunshot wounds were not so often. A great number of traumatic amputations of extremities were daily dressed, then every 2, and finally every 5 days. We had to kneel down very often while changing the dressing, since the mattresses were tightly lined up very close to each other on the floor. The most severely wounded were more frequently dressed. Physiotherapists, who performed exercise with all the wounded, worked with us.

## The dead

Initially, the dead were buried in the town cemetery. With time, however, the number of the dead persons increased and the transport to the cemetery became impossible. The majority of the killed persons were civilians. During the last 10 days, it was impossible to bury the dead, so about 100 dead bodies lay, designated with numbers, wrapped in plastic bags, in the yard across the Hospital.

## Conclusion

The relationship among the doctors who stayed in the hospital was correct. All the auxiliary and medical staff members did their job professionally and conscientiously. However, the information leaked out: everything repaired and put into function would be targeted and destroyed soon thereafter, the more so if it was of supreme importance for hospital function (aggregates, water supply, kitchen, sterilization units, wash-rooms, bakery). Later, a part of the hospital staff stayed in the hospital with the YFA. The moral of the wounded was very high indeed. Poor working conditions did not substantially influence the level of patient care and treatment. The enemy could not kill our affection for fellow human beings and our devotion to life.

